# Anatomy‐based definition of point A utilizing three‐dimensional volumetric imaging approach for high‐dose‐rate (HDR) intracavitary brachytherapy dose prescription when treating cervical cancer using limited resources

**DOI:** 10.1120/jacmp.v17i6.6029

**Published:** 2016-07-16

**Authors:** Manish K. Goyal, D.V. Rai, Than S. Kehwar, Jayanand Manjhi, Bret H. Heintz, Kathleen L. Shide, Jerry L. Barker

**Affiliations:** ^1^ Department of Radiation Oncology Texas Oncology Fort Worth TX USA; ^2^ Shobhit University Meerut UP India; ^3^ Department of Radiation Oncology Pinnacle Health Cancer Center Harrisburg PA USA

**Keywords:** high‐dose‐rate brachytherapy, cervical cancer, American Brachytherapy Society, point A, Groupe européen de curiethérapie — European Society for Therapeutic Radiology and Oncology

## Abstract

This study was designed to determine whether volumetric imaging could identify consistent alternative prescription methods to Manchester/point A when prescribing radiation dose in the treatment of cervical cancer using HDR intracavitary brachytherapy (ICBT). One hundred and twenty‐five treatment plans of 25 patients treated for carcinoma of the cervix were reviewed retrospectively. Each patient received 5 fractions of HDR ICBT following initial cisplatin‐based pelvic chemoradiation, and radiation dose was originally prescribed to point A (ICRU‐38). The gross tumor volume (GTV) and high‐risk clinical target volume (HR‐CTV) were contoured in three dimensions on the CT datasets, and inferior–superior, anterior–posterior, and left–right dimensions HR‐CTV were recorded along with multiple anatomic and skeletal dimensions for each patient. The least square–best fit regression lines were plotted between one half of the HR‐CTV width and pelvic cavity dimension at femoral head level and at maximum cavity dimension. The points in both plots lie reasonably close to straight lines and are well defined by straight lines with slopes of 0.15 and 0.17; intercept on y‐axes of ‐0.08 and ‐0.03, point A, at the same level as defined based on applicator coordinates, is defined using this correlation, which is a function of distance between femoral heads/dimensions of maximum pelvic cavity width. Both relations, defined by straight lines, provide an estimated location of point A, which provides adequate coverage to the HR‐CTV compared to the point A defined based on applicator coordinates. The point A defined based on femoral head distance would, therefore, be a reasonable surrogate to use for dose prescription because of subjective variation of cavity width dimension. Simple surrogate anatomic/skeletal landmarks can be useful for prescribing radiation dose when treating cervical cancer using intracavitary brachytherapy in limited‐resource settings. Our ongoing work will continue to refine these models.

PACS number(s): 87.55.D‐, 87.55.ne

## I. INTRODUCTION

Intracavitary brachytherapy (ICBT) plays a critical role in the curative treatment of cervical cancer. Multiple studies have demonstrated a decrease in local recurrence and an improvement in overall survival when brachytherapy is a component of definitive radiation treatment.^1^, ^2^, ^3^, ^4^ The success of brachytherapy requires extreme conformity, with the delivery of a high radiation dose directly to the tumor while sparing surrounding normal tissues via rapid radiation dose falloff beyond the implanted tumor volume.

Classically, several dosimetry systems were designed to guide implant procedures and report dose specification for brachytherapy treatment of cervical cancer. The Manchester dosimetry system is one of the most extensively used in clinics worldwide due to its simplicity and reproducibility. It was designed by Merdith and Messey in the 1940s, and popularized in the era of two‐dimensional radiotherapy imaging and treatment planning.^5^, ^6^ Nevertheless, this system and its variants continue to be used clinically worldwide, particularly in radiation oncology settings with limited technological resources. In the Manchester system, orthogonal X‐ray radiographs of the pelvis are used to define reference points, such as “point A,” to prescribe and report the radiation dose. Point A was originally defined as a point located 2 cm superior to the lateral vaginal fornix and 2 cm lateral to the cervical canal, assuming that the region represented the tolerance limits due to crossing of the uterine artery and ureter. This definition was later modified as a point located 2 cm superior to the external cervical os and 2 cm lateral to the cervical canal.^5^, ^6^ The International Commission on Radiation Units and Measurements Report Number 38 (ICRU‐38) discussed dose and volume specifications for reporting intracavitary brachytherapy^(7)^ and is widely accepted clinically. Technological development has resulted in the ability to use higher‐activity miniature radioactive sources in brachytherapy with added advantages of rigid immobilization, outpatient treatment, patient convenience, accuracy of source and applicator positioning, treatment optimization and radiation protection for personnel involved.^8^, ^9^, ^10^, ^11^ Originally, point A was defined for dose prescription of low‐dose‐rate (LDR) intracavitary brachytherapy but has been successfully used for dose prescription for HDR‐ICBT.^9^, ^10^, ^11^

With advances in imaging technology and three‐dimensional (3D) treatment planning systems, clinical dose prescription is transitioning away from 2D‐Manchester approaches toward 3D‐volumetric approaches where adequate dosimetric coverage of a clinical target volume (CTV) is evaluated.^(12)^ The assumption of point A representing the crossing of the uterine artery and ureter is questionable because it was seen that the mean distances between the brachytherapy point A (defined based on applicator geometry) and anatomical point A (crossing of the uterine artery and ureter) were 5.2 cm (SD: ± 1.0) on right and 5.4 cm (SD: ± 1.1) on left and, furthermore, the dose received to the anatomical point A on right and left were 35.2% and 30% of the doses prescribed to the right and left brachytherapy point, respectively, which demonstrates that location of point A does not always represent the crossing point of uterine artery and ureter.^(13)^ The limitations of orthogonal radiographs and dose prescription on point A warrant expedition of 3D brachytherapy based on CT/MRI planning.^(14)^ A proposed goal for 3D image‐guided conformal brachytherapy treatment is for 90% of the high‐risk clinical target volume $\left(\text{HR}‐\text{CTV}\;D_{90}\right)$ to receive the prescribed dose.^(15)^ In 2000, Groupe européen de curiethérapie — European Society for Therapeutic Radiology and Oncology (GEC‐ESTRO) created a working group and decided to support 3D imaging based 3D treatment planning approach in cervix cancer brachytherapy.^(16)^ The second part of the GEC‐ESTRO working group presented the formulated recommendations transiting from traditional 2D approach to 3D image‐based therapy for cervix cancer.^(17)^ American Brachytherapy Society (ABS) presented its recommendations in 2011, published in 2012, and supported GEC‐ESTRO guidelines.^18^, ^19^ However, ABS and GEC‐ESTRO continue to recommend the recording of conventional point A doses during 3D‐image based treatment planning, at least during this ongoing transition period.^14^, ^19^

Volumetric imaging may not be feasible for clinics that either do not have onsite CT/MRI imaging or when patient refuses volumetric imaging due to any reason (i.e., personal, financial, or any unknown reason). In this study, an alternative method for dose prescription is investigated by evaluating retrospective patients who received HDR‐ICBT, and lateral position of point A was defined without changing superior location. A revised anatomy‐based point A definition is presented utilizing a three‐dimensional volumetric imaging approach. A dose prescription to revised point A will provide the adequate coverage to tumor considering HR‐CTV volume for the respective pelvic cavity size.

## II. MATERIALS AND METHODS

### A. Patient selection

Twenty‐five retrospective patients with carcinoma of the cervix, treated with HDR brachytherapy from January 2009 to January 2013, are included in this study. These patients had pathologically proven locally advanced (FIGO stage IB or higher) squamous cell carcinoma or adenocarcinoma of the uterine cervix, and were treated with external beam radiation therapy (EBRT) to a dose of 45 Gy in 25 fractions, 5 fractions per week over a period of five weeks to the whole pelvis, with concurrent cisplatin‐based chemotherapy. Near the completion of EBRT, 5 fractions of HDR‐ICBT were delivered by Varisource HDR brachytherapy Ir‐192 remote afterloader (VariSource, Varian Medical Systems, Palo Alto, CA) using a CT‐MR compatible Fletcher applicator set (tandem/ovoid or tandem/ring) and radiation dose was prescribed to point A. Dose to point A was in the range of 4.0–6.0 Gy per fraction. Treatment planning for all the patients was performed using a volumetric CT dataset obtained for each brachytherapy fraction imported into a treatment planning system (TPS) (BrachyVision, Varian).

### B. ICBT implant

The first ICBT implant was performed with general anesthesia in the hospital's operating room by the radiation oncologist. A Smitt sleeve was implanted during first ICBT procedure to facilitate subsequent outpatient treatment. Anterior and posterior vaginal packing was used during each implant to displace bladder anteriorly and rectum posteriorly to further minimize doses to bladder and rectum.

### C. CT acquisition/patient preparation

All patients underwent pelvic CT scan following ICBT implant using helical scanning with slice thickness of 3 mm. A Foley catheter with the balloon insufflated with 7 cc of radiopaque contrast material was used for determination of an ICRU bladder point. CT datasets were acquired such that the scan would include at least 3–4 cm margin superior to the proximal tandem position and to the entire implant inferiorly.

### D. Treatment planning

All the patients were planned using ICRU‐38 guidelines and dose was prescribed to point A. For the purposes of this study, the treatment plans were subsequently reanalyzed with high‐risk clinical target volumes (HR‐CTVs) and organs at risk (OAR) (i.e., the rectum and the bladder) contoured for each CT dataset from each treated fraction by the attending physician involved in the original case. Preset pelvis window/leveling and CT parameters were used to maintain consistent contouring conditions for all treatment plans. The entire bladder wall and rectum were contoured, with the bladder wall including the balloon with contrast and the rectum contoured from anorectal to recto–sigmoid junction. The sigmoid colon was contoured from the recto–sigmoid junction to about 2 cm above the tip of the central tandem. Care was taken to insure that the sigmoid was contoured adjacent to or above the uterus near the implanted brachytherapy applicator, when applicable.

HDR brachytherapy applicators included tandem/ovoid or tandem/ring. Tandem sizes were 4, 6, or 8 cm with angles of 15°, 30°, 45°, or 60°. Ovoid sizes included mini, small, medium, and large with the buildup diameter of 1.6, 2.0, 2.5, or 3 cm, respectively. Ring angles included 30°, 45°, or 60°. Ring applicators had two possible buildup caps of 5 mm or 7.5 mm anterior–posteriorly with 5 mm buildup laterally. Applicators were defined in the TPS and evaluated using 3D display tools. Initially, sources were loaded using department protocol and then were modified using a graphical dose shaper or with iterative/manual adjustment of individual HDR source dwell positions to optimize the dose to point A and to OARs.

### E. Data collection and analysis

**Figure 1:**
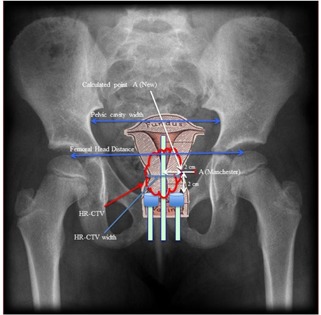
Criteria to measure different dimensions are shown as pelvic cavity width, femoral head distance, HR‐CTV width, and positions of Manchester point A and new point A.

Since each of the 25 patients received five separate HDR ICBT applications, a total of 125 treatment plans were reviewed and analyzed in this study. In each of the 125 plans, radiation dose was originally prescribed to point A (ICRU‐38), and tumor volumes and high‐risk clinical target volumes (HR‐CTV) were retrospectively redrawn for all plans. The maximum width of HR‐CTV, in left‐right directions, distance between femoral heads at the level of the mid femoral head, and maximum width of the pelvic cavity at midpelvis were measured for each plan, as shown in Fig. 1. The HR‐CTV volume and D90 dose was also recorded for all plans from respective DVH.

### F. Validation of the results

For validation of the finding of this study, another set of 25 additional patients, treated during June 2014 to Dec. 2015, were included. Each patient received 5 fractions of HDR and plans were generated for all 5 fractions. The HR‐CTV is drawn by the treating physician using similar approach to that used in first set of patients. Dose is prescribed to the calculated point A, which was defined using femoral head dimensions, and doses at the point A (Manchester) and the point A (ABS), and D90 were recorded for each plan.

## III. RESULTS

The values of D90, HR‐CTV volume, HR‐CTV width (in the left‐right dimension), femoral head distance, and pelvic cavity width were measured and averaged for the plans of 5 fractions of each patient. The D90 is normalized to the prescribed dose to eliminate prescription dose dependency of D90; that is to say, the normalized D90 (ND90) is defined as the ratio of D90 to the prescribed dose at point A. For testing the reliability of point A for dose prescription in the HDR intracavitary brachytherapy of carcinoma of the cervix, the HR‐CTV coverage is examined

**Figure 2:**
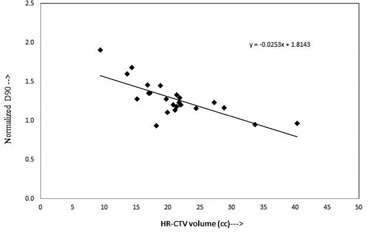
Plot between normalized D90 of HR‐CTV and volume of HR‐CTV represents a linear correlation.

**Figure 3:**
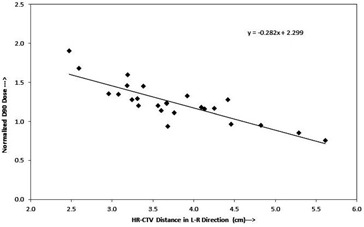
Plot between normalized D90 of HR‐CTV and left to right width of HR‐CTV shows a linear correlation.

Figures 2 and 3 represent the plots of ND90 versus HR‐CTV volume, and ND90 versus HR‐CTV width (left–right), for each of the datasets referred above, respectively. Both of the datasets plotted in these graphs appear to fit straight lines relatively well. In each case, there is a certain amount of scattering in the plotted points about the best‐fit regression lines with slopes of ‐0.0253 and ‐0.282, and vertical intercepts of 1.814 and 2.299, respectively. The Pearson correlation coefficient $r^{2}$ of 0.74 and 0.84, and $r^{2}$ of 0.54 and 0.71 for Figs. 2 and 3, respectively.

**Figure 4:**
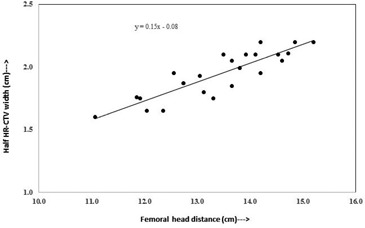
Plot between femoral head distance and half width of HR‐CTV in left to right direction at maximum width level.

**Figure 5:**
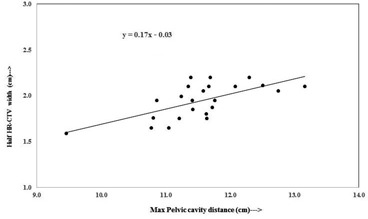
Plot between maximum pelvic cavity distance and half width of HR‐CTV in left to right direction at maximum width level.

Figures 4 and 5 show plots of one‐half of the HR CTV width versus distance between femoral heads, and one‐half of the HR‐CTV width versus maximum cavity dimension, respectively. Both datasets, plotted in Figs. 4 and 5, fit straight lines with slight scattering of the plotted points about the best‐fit regression lines. The regression lines fitted to these datasets have slopes of 0.15 and 0.17 with vertical intercepts of ‐0.08 cm and ‐0.03 cm. Pearson correlation coefficient $r^{2}$ of 0.88 and 0.66, and $r^{2}$ of 0.77 and 0.43, respectively. When the best‐fit regression lines are forced to pass through the origin, the slopes of the lines were obtained to be 0.14 cm and 0.17, respectively, and reasonably close to that of the best‐fit lines with vertical intercepts. In these plots, the outlier points (two outlier points in each figure) were not accounted.

From Figs. 4 and 5, point A, at the same level as defined based on applicator coordinates, can be defined by the correlations provided below.

Point A based on distance between femoral heads (Fig. 4):

A(cm)=0.15×(R−Ldist.between Fem. Head in cm)−0.08

Point A based on dimension of maximum pelvic cavity widths (Fig. 5):

A(cm)=0.17×(Max Pelvic Cavity width in cm)−0.03

These relations give fairly appropriate location of point A, which provides adequate coverage to the HR‐CTV compared to the point A defined based on applicator coordinates.

The cavity width measurements at maximum dimension are subjective and may have variation from patient to patient, while the femoral head distance measurements are reproducible and can be measured with least variation. Hence, the point A defined based on femoral head distance would be an appropriate tool to use for dose prescription.

There was a statistically significant difference between ND90 of first set of patients where dose was prescribed to point A (Manchester) and ND90 of second set of patients where dose was prescribed to calculated point A (p = 0.0007, Student's *t*‐test).

**Figure 6:**
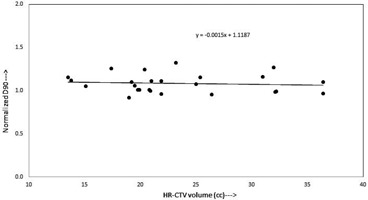
Plot between normalized D90 of HR‐CTV and HR‐CTV volume (cc) represents a linear correlation when dose is prescribed to the calculated point A using Eq. (1).

Figure 6 represents the plot of normalized D90 (ND90) versus HR‐CTV volume, for second set of plans, where dose is prescribed to the calculated point A. The dataset plotted in the graph appears to fit a straight line relatively well, where there is a certain amount of scattering in the plotted point about the best‐fit regression line with a slope of ‐0.0015, and vertical intercept of 1.1187, which are not different than 0 and 1, respectively.

## IV. DISCUSSION

At present, in many radiation therapy centers, the HDR ICBT dose is prescribed to point A, which is defined on the basis of applicator position. This practice is meaningful only if the HR‐CTV width is encompassed within bilateral point As (i.e., left point A and right point A). The anatomic significance of point A has been questioned by several investigators.^11^, ^14^, ^19^ Point A relates to the positions of the sources and not to a specific anatomic structure and, depending on the size of the cervix point A, may lie inside or outside of the tumor. Thus, dose prescription at point A could risk underdosage of large cervical cancers or overdose of small ones^(14)^ which is clear in Fig. 2, where the ND90 decreases from 1.90 to 0.93 with an increase of HR‐CTV volume from 9.43 cc to 40.32 cc. Similarly, in Fig. 3 the ND90 decreases with an increase in HR‐CTV width (left‐right) at the level of point A, while in Fig. 6 it is seen that ND90 is almost constant for all HR‐CTV volumes, where dose is prescribed to the calculated point A, which ensures adequate coverage of the HR‐CTV. When dose is prescribed to calculated point A, which is a function of femoral head distance, the dose to point A (Manchester/ABS) may be lower or higher depending on the location of calculated point A.

Lindegaard et al.^(20)^ reported that the D90 decreases with the increase of HR‐CTV volume. In this study, 3D treatment plans of 72 locally advanced carcinoma of the cervix were evaluated, where it is shown that the image‐based DVH analysis of standard point A prescription resulted highly variable tumor doses ranging from 52%–160% of the prescription dose.^(21)^ In another study, Anderson et al.^(22)^ evaluated point A doses of 55 HDR plans of 36 patients, using conventional and MRI‐guided 3D conformal plans, and their results indicate that the variation of point A doses was up to 11%–12% compared to conformal plans.

## V. CONCLUSIONS

The results of the present study provide a hybrid approach, where the outcome of 3D planning is extended to use into a 2D environment, potentially useful for facilities with limited resources. The GEC‐ESTRO and the ABS guidelines were used to generate 3D datasets, including patients’ anatomical information, and treatment plans of retrospective HDR patients, and a new approach for defining point A was derived. Femoral head distance and maximum pelvic cavity dimension are the fixed anatomical geometry for an individual patient and can easily be recorded from 2D radiographs and consequently can be used in defining point A.

Results of this study reveal that the dose prescription to classically defined point A may underdose or overdose the tumor due to variable size and anatomy of the patients. The mathematical relationships derived in this study could be a valuable tool for centers with limited resources (i.e., without 3D imaging facilities). These centers can use a relation using 2D radiographs to define point A for each patient, which will be based on the individual patient's anatomy. Dose could then be prescribed to a modified point A to obtain proper tumor coverage.

Based on the results of this study, it is concluded that the point A defined based on femoral head distance or pelvic cavity depth gives promising coverage to the cervix irrespective of individual patient anatomical variation, compared to traditionally defined point A. We, however, like to caution users to test this proposed model on previously treated patients in their institutions to fully understand the concept prior to clinically utilization of the model. Our ongoing work will continue to refine these models.

## COPYRIGHT

This work is licensed under a Creative Commons Attribution 3.0 Unported License.

## Supporting information

Supplementary Material

Supplementary Material

Supplementary Material

Supplementary Material

Supplementary Material

Supplementary Material

Supplementary Material

Supplementary Material

Supplementary Material

Supplementary Material

Supplementary Material

Supplementary Material

Supplementary Material

Supplementary Material

Supplementary Material
