# Supplementary figures and images for: Anatomy‐based definition of point A utilizing three‐dimensional volumetric imaging approach for high‐dose‐rate (HDR) intracavitary brachytherapy dose prescription when treating cervical cancer using limited resources
Source: J Appl Clin Med Phys. 2016 Jul 16;17(6):69–77. doi: 10.1120/jacmp.v17i6.6029 (PMC5690503; doi:10.1120/jacmp.v17i6.6029)

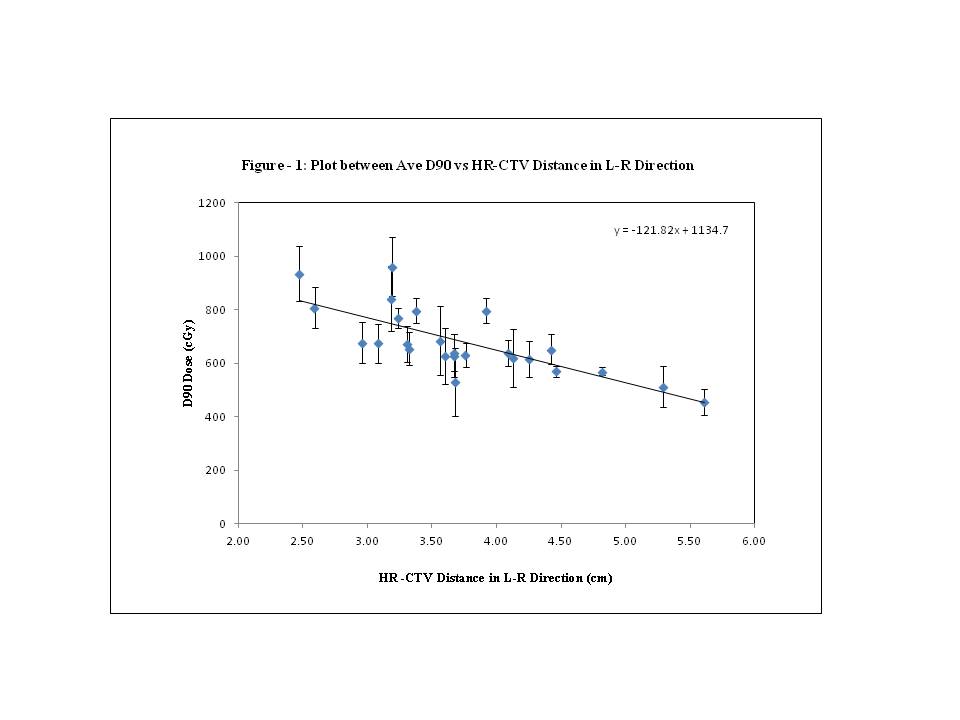

Supplement: Supplementary file 1 — Supplementary Material [file ACM2-17-069-s001.jpg]

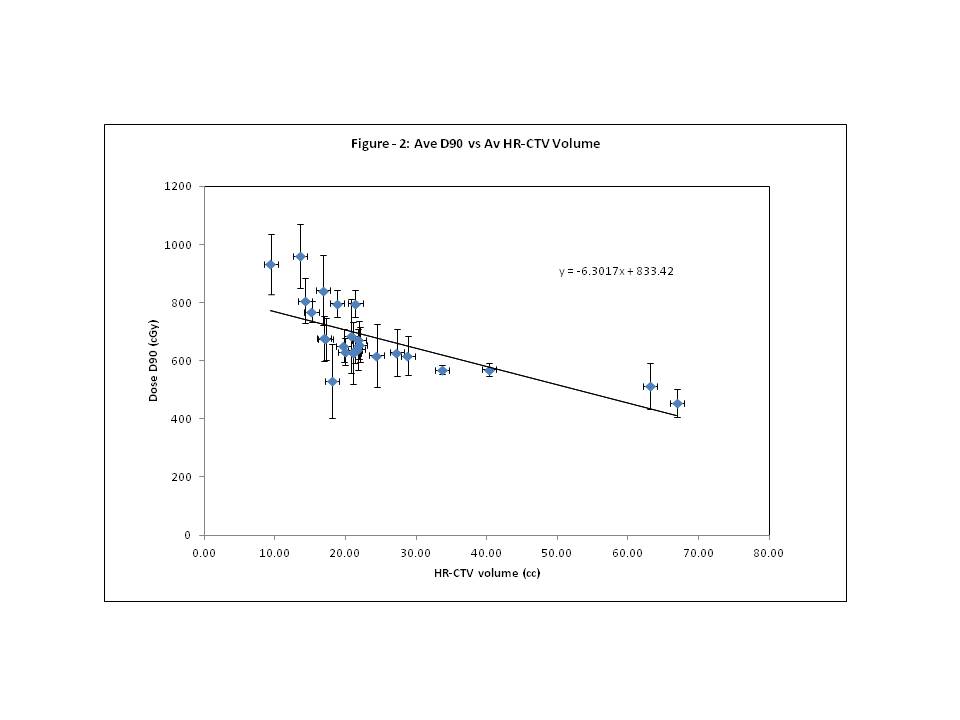

Supplement: Supplementary file 2 — Supplementary Material [file ACM2-17-069-s002.jpg]

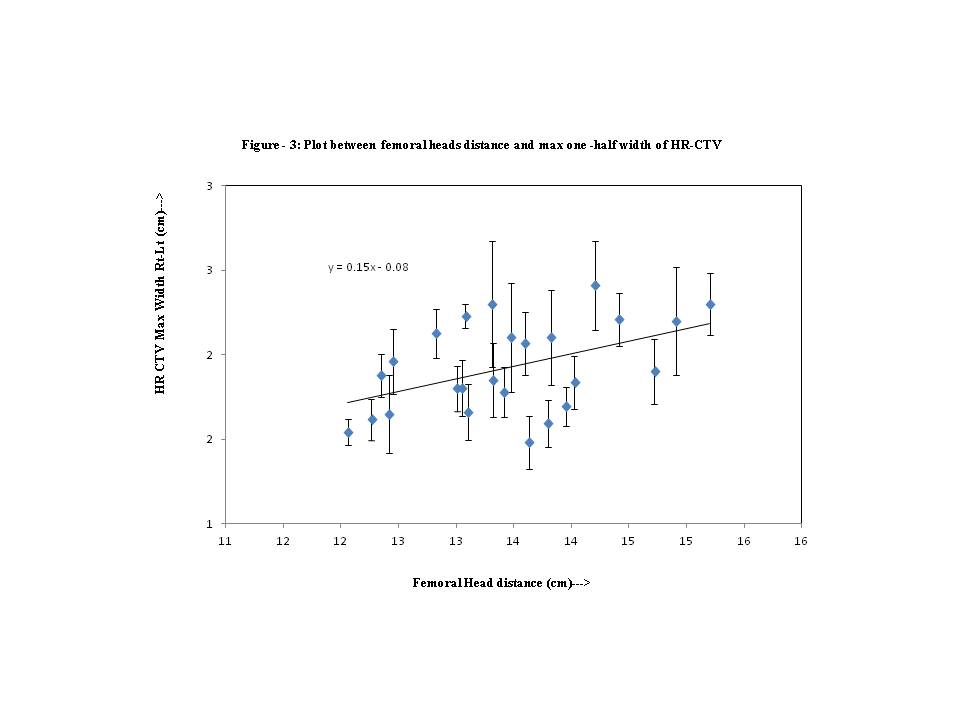

Supplement: Supplementary file 3 — Supplementary Material [file ACM2-17-069-s003.jpg]

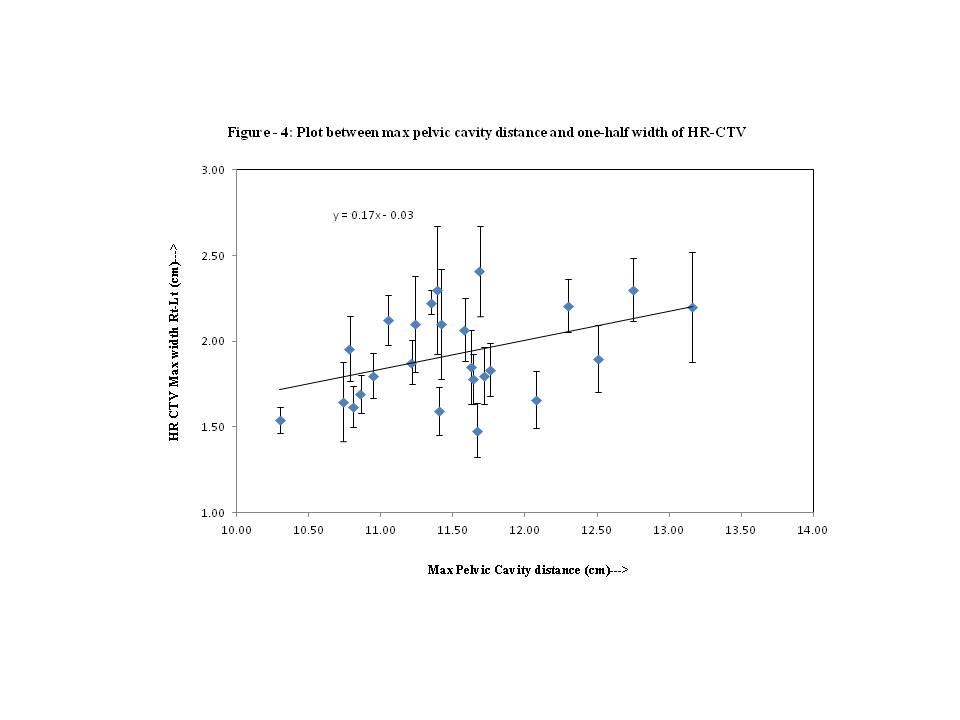

Supplement: Supplementary file 4 — Supplementary Material [file ACM2-17-069-s004.jpg]

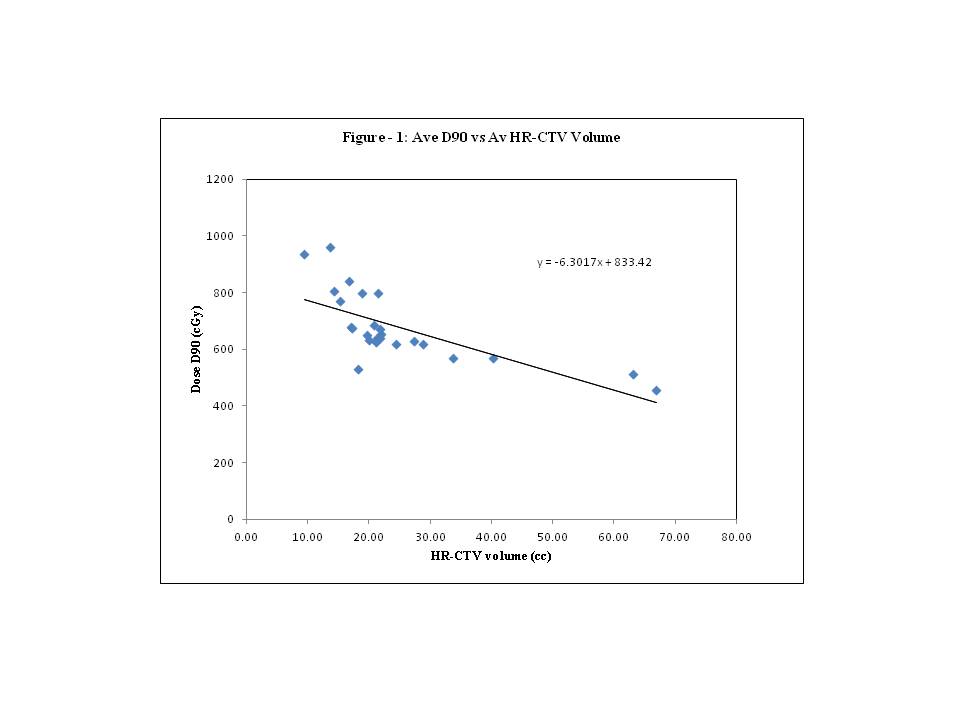

Supplement: Supplementary file 5 — Supplementary Material [file ACM2-17-069-s005.jpg]

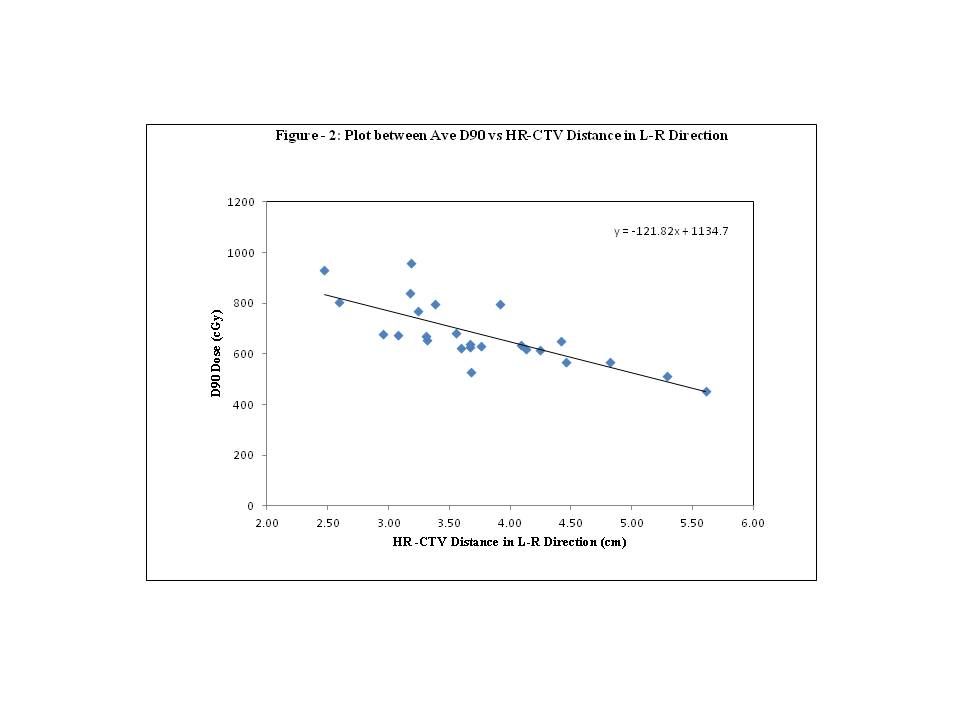

Supplement: Supplementary file 6 — Supplementary Material [file ACM2-17-069-s006.jpg]

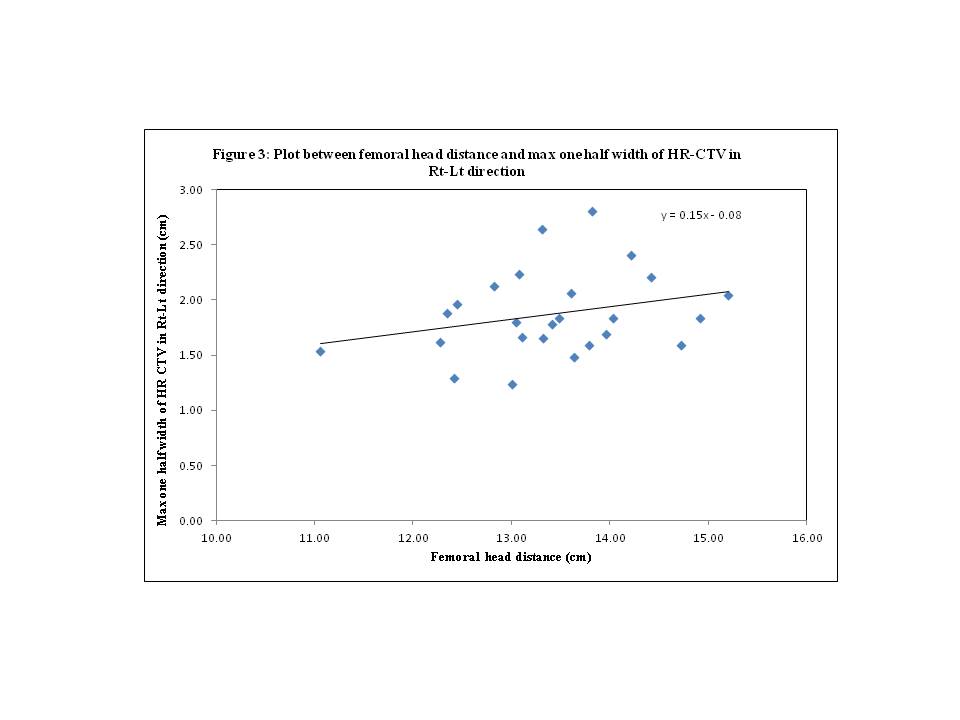

Supplement: Supplementary file 7 — Supplementary Material [file ACM2-17-069-s007.jpg]

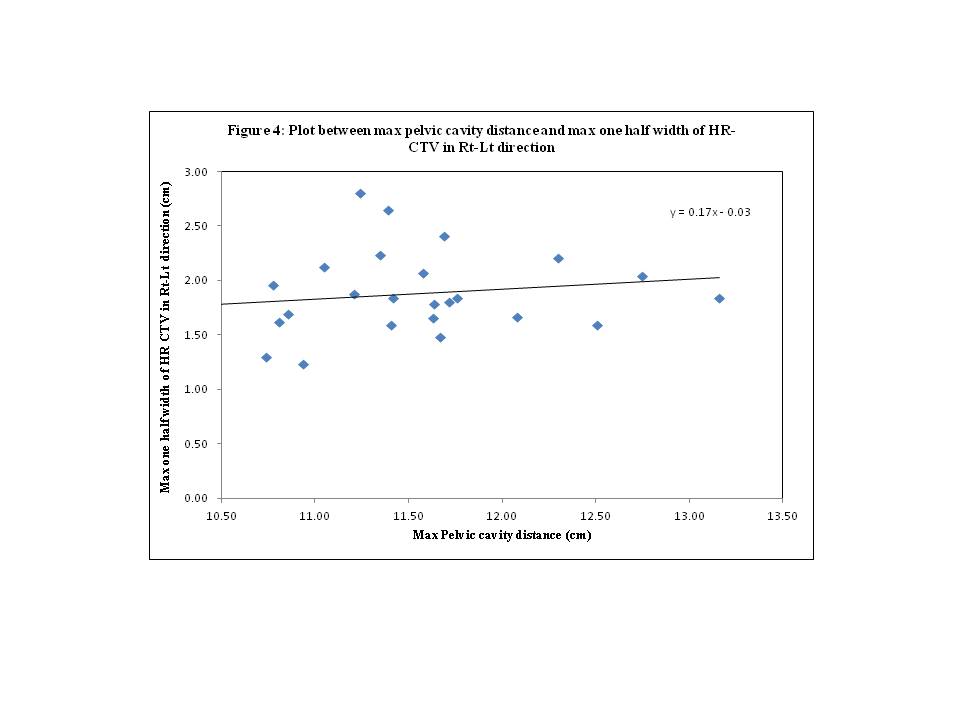

Supplement: Supplementary file 8 — Supplementary Material [file ACM2-17-069-s008.jpg]

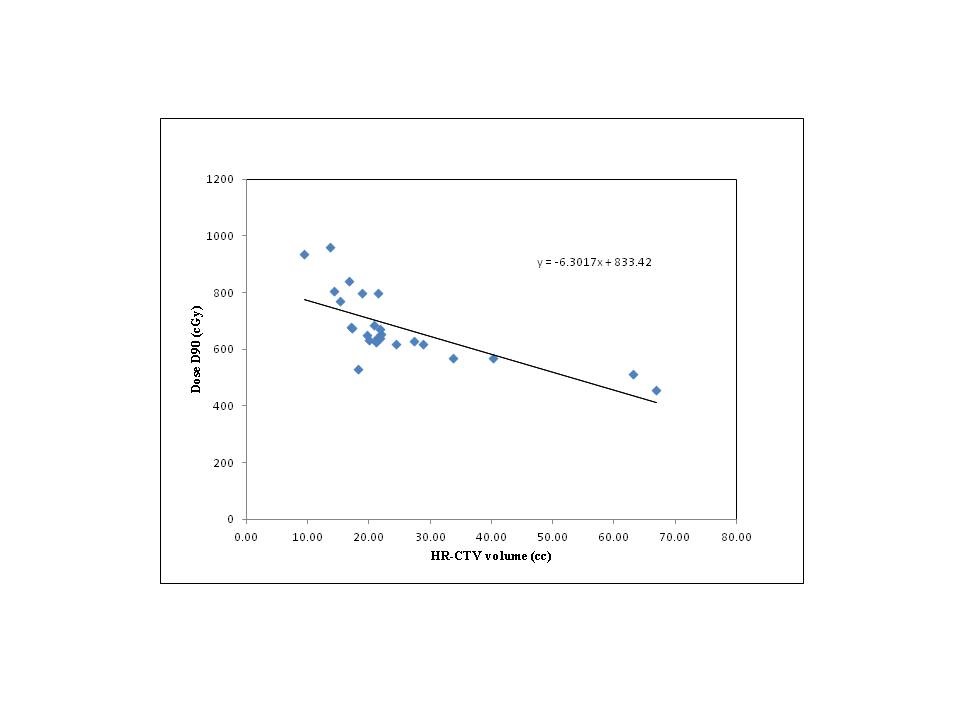

Supplement: Supplementary file 9 — Supplementary Material [file ACM2-17-069-s009.jpg]

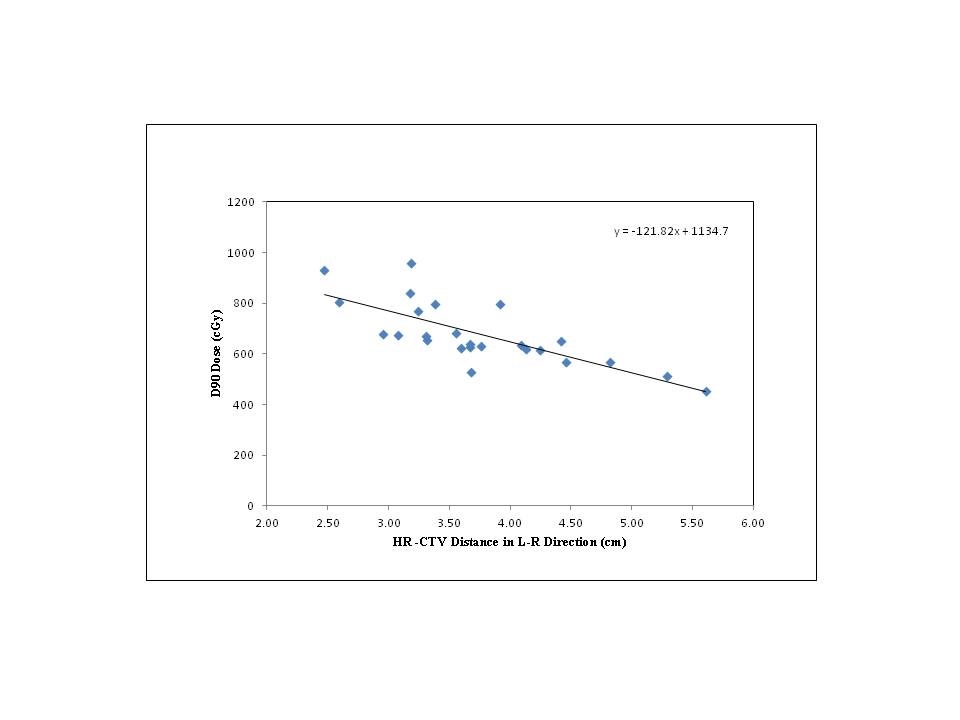

Supplement: Supplementary file 10 — Supplementary Material [file ACM2-17-069-s010.jpg]

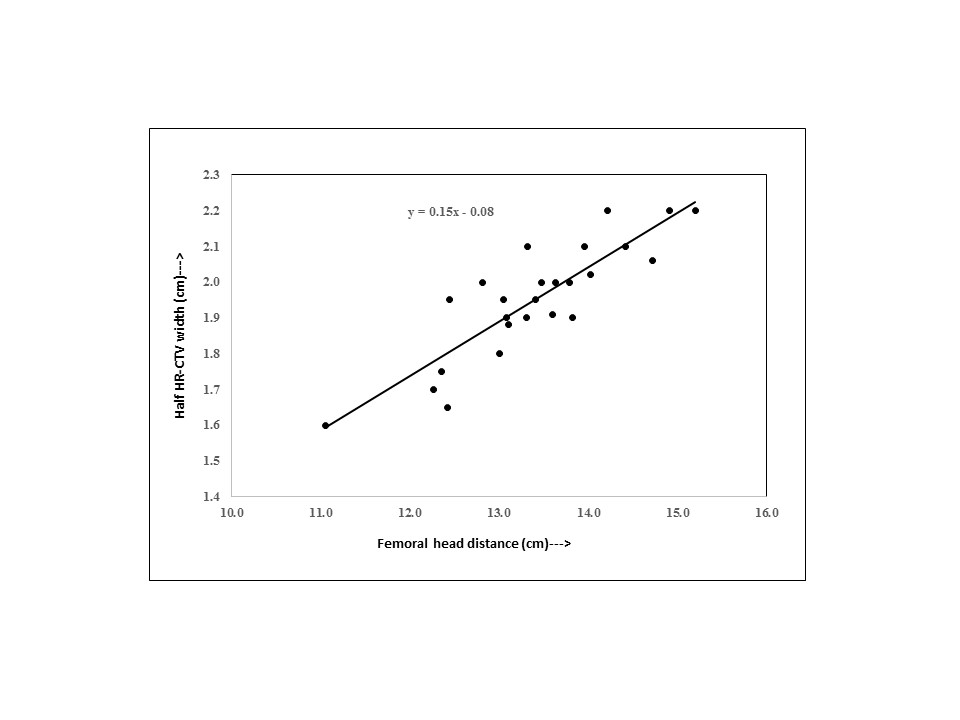

Supplement: Supplementary file 11 — Supplementary Material [file ACM2-17-069-s011.jpg]

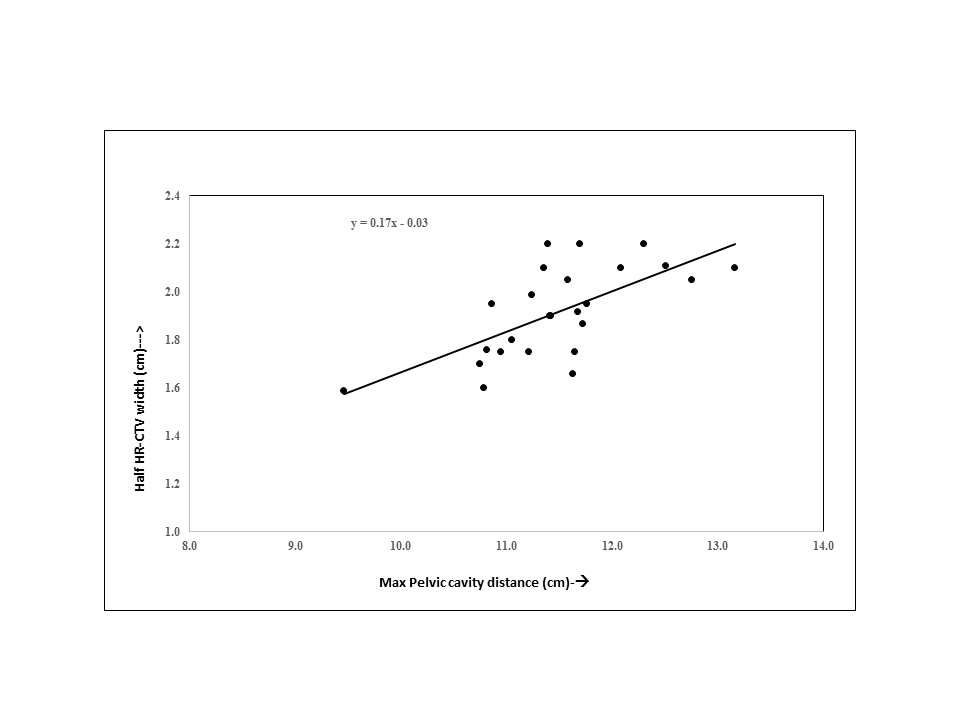

Supplement: Supplementary file 12 — Supplementary Material [file ACM2-17-069-s012.jpg]
